# Fractal, Multifractal, and Lacunarity Analysis of Microglia in Tissue Engineering

**DOI:** 10.3389/fbioe.2015.00051

**Published:** 2015-04-14

**Authors:** Audrey L. Karperien, Herbert F. Jelinek

**Affiliations:** ^1^Centre for Research in Complex Systems, School of Community Health, Charles Sturt University, Albury, NSW, Australia

**Keywords:** microglia, fractal analysis, multifractal, lacunarity, tissue engineering

## Introduction

Tissue engineering is currently one of the most exciting fields in biology (Grayson et al., 2009). Fractal analysis is equally exciting (Di Ieva et al., 2013), as is the study of microglia, the brain’s immuno-inflammatory cell, recently shown to be of considerably more importance than previously imagined in both healthy and diseased brain (Tremblay et al., 2011). Each of these fields is developing at a pace far outstripping our capacity to integrate and translate the information gained into clinical use (Karperien et al., 2008b, 2013; Jelinek et al., 2011, 2013), and the excitement more than trebles where these fields intersect. Three elements of fractal analysis – monofractal, multifractal, and lacunarity analysis – applied to microglia may contribute significantly to the next steps forward in engineered tissues and 3D models in neuroscience.

### Fractal analysis and lacunarity

To define “fractal analysis” would take a volume, but for this commentary, it is sufficient to understand that fractal analysis in biology assesses the scaling inherent in biological forms or events, and turns out a statistical index of complexity having no units called the “fractal dimension” (*D*_F_). This number measures not length, width, height, or density, but scale-invariant detail. For a pattern to have fractal scale-invariant detail means that the pattern repeats itself infinitely as one inspects it at closer and closer resolution (magnifies it), where that detail is not trivial. To elaborate, as one magnifies a simple line, it infinitely repeats itself quite trivially as a simple line, but as one magnifies a fractal line, one finds it never resolves into straight pieces but rather each magnified segment repeats the initial fractal pattern infinitely. A *D*_F_ measures this infinite scaling, quantifying complex patterns without rendering meaningless the relative numbers of large and small measurements within them. Without getting too technical, fractal analysis of a simple line yields a *D*_F_ of 1.00, and the higher the “complexity,” the higher the *D*_F_ (Mandelbrot, 1983; Takayasu, 1990). Building on this so-called monofractal analysis, *multifractal* analysis, to summarize, is a way of finding for a single pattern a spectrum of *D*_F_s, owing to a pattern having characteristically multiple degrees of scaling, such as could be imagined for a cascading fractal phenomenon (Jestczemski and Sernetz, 1996; Falconer, 2014).

The word “lacuna” is derived from the word for lake, and refers to a *gap* or *pool*. In fractal analysis, lacunarity translates to measures of gappiness or “visual texture,” such as might be seen in the patchiness of forests, for instance (Plotnick et al., 1993). It has been defined as the degree of *inhomogeneity* and *translational and rotational invariance* in an image (Plotnick et al., 1993; Smith et al., 1996), where low lacunarity implies homogeneity and that rotating the image will not change it significantly. Thus, an image having mostly similarly sized gaps and little rotational variance would be expected to have low lacunarity, and one with much heterogeneity, many differently sized gaps, and notable rotational variance, would be expected to have high lacunarity (Karperien et al., 2011a). Lacunarity is frequently assessed during fractal analysis because the data on which it is based are easily collected by the same methods. The details and calculations behind fractal analysis are beyond the scope of this commentary but user-friendly, freely available software for biologists (Karperien, 2001, 2013) and in-depth explanations are available elsewhere (Smith et al., 1996).

### Microglia

Microglia are of considerable interest to the tissue engineer interested in the central nervous system (CNS). These are tiny immuno-inflammatory cells that are very abundant in and wield considerable power in the brain and spinal cord of humans as well as many other species (Dowding and Scholes, 1993; Sheffield and Berman, 1998; Bernhardi and Nicholls, 1999; Sierra et al., 2014a). They are considered structural in some senses, and are indeed immune cells, yet traffic through the CNS, and are not grossly separated from their surroundings in the way that the meninges can be peeled from the brain or lymph nodes are segregated from surrounding tissue, for instance. Similar in number to neurons but much smaller in size, microglia in living organisms are usually found as individual cells physically integrated within the tangled mesh of cells that is the CNS (Lawson et al., 1990; Rezaie and Male, 1999; Billiards et al., 2006; Inoue, 2006; Stoll et al., 2006; Leung et al., 2008; Morgan et al., 2012; Zhao et al., 2012; Hinwood et al., 2013).

They play key roles in immature, developing nervous tissue, and in adult tissue, they ensure normal goings on but also police, protect, repair, and remodel neurons, including by removing cell parts and debris (Sierra et al., 2014a,b). They are meaningfully involved in virtually everything that goes on in the brain, from mediating behavioral effects of emotional stress (Hinwood et al., 2013) to autism (Maezawa et al., 2011; Morgan et al., 2012) to cleaning up after a stroke (Vinet et al., 2012). The scientific community has show-cased them using time-lapse photography and *in vivo* thin-skull visualization, revealing how they move within their space, by furling, unfurling, and waving their processes about, and throughout their space, migrating and phagocytosing (Nimmerjahn et al., 2005; Tremblay et al., 2011). Marvelously, they have no single form, rather, they exist along a highly disparate continuum of forms, shape-shifting to meet the most immediate challenge to the neurons they support, morphing back and forth as required (Karperien et al., 2013). Indeed, their function is usually inferred largely from their form, albeit generally backed up with biochemical and other data (Streit and Kreutzberg, 1987; Kreutzberg, 1995; Banati et al., 1999; Orlowski et al., 2003; Sheets et al., 2013).

## Measuring Microglia with Fractal Analysis

**Figure 1:**
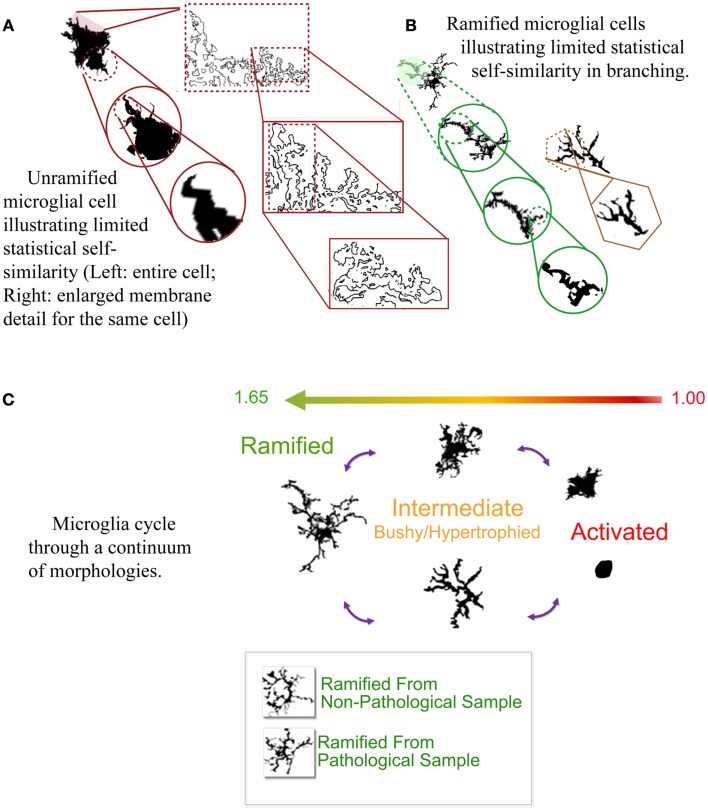
**(A,B)** Self-similarity in two typical microglial morphologies. **(C)** The cycle of microglial morphology. Microglia adopt morphologies along a cycle that corresponds to their box-counting *D*_F_ (shown ranging from 1.00 to 1.65 in the figure). Ramified morphologies are more complex, and the most activated, rounded forms least complex. The bottom of **(C)** shows two cells from pathological vs. non-pathological tissue that were visually indistinguishable but objectively distinguishable by the box-counting *D*_F_. Author’s figures adapted from Karperien et al. (2013).

What is perhaps most marvelous of all is that their morphology can be measured by their *D*_F_, as well as their lacunarity, and to some extent multifractal spectra (Soltys et al., 2001; Jelinek et al., 2008, 2011; Karperien et al., 2008c, 2011b, 2013). Finding this was a relief to the beleaguered microgliologist, because microglial morphology is not easily quantifiable by traditional measures despite that microglial function is so well-correlated with that morphology. Basically, while microglia change shape back and forth from highly ramified usually radially branched structures to plump and rounded blobs, their *D*_F_s range from higher to lower values corresponding to the spectrum of morphological change (see Figure 1) (Karperien et al., 2013). Results of *in silico* modeling studies agree with these general conclusions from studies of actual cells, showing microglia can be successfully modeled using sets of increasingly complex fractal branching parameters (Jelinek et al., 2002; Jelinek and Karperien, 2008).

The practical value of fractal analysis surpasses classifying individual cells and verifying models. The *D*_F_ has been used to analyze overall status in pathological conditions and aging (Jelinek et al., 2008; Karperien et al., 2008a,c). Data from biological and *in silico* cells (Jelinek et al., 2002) suggest the significance of multifractal scaling in particular is that it identifies microglia in temporarily hyper-ramified transitional states between ramified and intermediately activated forms.

Lacunarity, like multifractal spectra, also complements the *D*_F_. Lacunarity and *D*_F_s have been shown to be correlated in some research, but not by all methods of fractal analysis. For microglia, the box-counting *D*_F_ and lacunarity both generally decrease as cells cycle toward a more activated state, then increase as they return to a ramified state (Jelinek et al., 2008), but this is not strictly the case and the exceptions are meaningful. It has been established using box-counting fractal analysis methods that some patterns indistinguishable by their *D*_F_s are distinguishable by their lacunarity, or vice versa, and such is the case for microglia (Karperien et al., 2011a, 2013). *In silico* modeling of microglia has shown that although the *D*_F_ is generally more sensitive using whole cells, lacunarity is more sensitive to changes in particular features such as soma size relative to process length (Karperien et al., 2011a, 2013). Lacunarity has also been demonstrated to better identify microglia than does the *D*_F_ in certain situations (e.g., elderly human cortex but not tumor) (Soltys et al., 2005; Karperien et al., 2011a).

## Conclusion

To sum up, our point here is twofold: first, to let the tissue engineer modeling CNS know that he or she needs to consider microglia, because despite that these cells are tiny and were once considered negligible for normal function, they are entirely engaged physically and physiologically within the CNS; and second, to ensure that he or she is aware that these cells are characterized by some degree of fractal scaling. When developing methods to restore and replace diseased tissue, the tissue engineer who does not consider these two factors may develop models that overlook or misrepresent events. In particular, there is a need to ensure that models, such as engineered tissues being used as 3D *in vivo* models and cell-culture models being used for things like pharmaceutical research, do not overlook ostensibly subtle features of microglial activity that are characterizable by fractal measures but not traditional measures, and may be very important (Leung et al., 2008; Katari et al., 2014). The work discussed here focused on individual cell changes, but such changes can be understood within broader notions of decreasing complexity with increasing pathology, perhaps attributable to decreasing ability to generate novel responses to deal with rapidly changing environments. At any rate, for engineering and modeling CNS, from cell-culture environments to tissue formation and function, microglia are tiny but critical components, and their fractal and multifractal features need to be considered.

## Conflict of Interest Statement

The authors declare that the research was conducted in the absence of any commercial or financial relationships that could be construed as a potential conflict of interest.
